# Singlet and triplet harvesting enable efficient NIR-II quantum-dot electroluminescence

**DOI:** 10.1093/nsr/nwaf552

**Published:** 2025-12-04

**Authors:** Wan-Shan Shen, Sam Teale, Yang Liu, Jia-Lin Pan, You-Jun Yu, Chen Zou, Feng Zhao, Hong-Wei Duan, Ye Wang, Zong-Shuo Liu, Hua-Hui Li, Patrick Knowels, Zeke Liu, Ya-Kun Wang, Henry J Snaith, Liang-Sheng Liao

**Affiliations:** Institute of Functional Nano & Soft Materials (FUNSOM), Jiangsu Key Laboratory for Carbon-Based Functional Materials & Devices, State Key Laboratory of Bioinspired Interfacial Materials Science, Soochow University, Suzhou 215123, China; Clarendon Laboratory, University of Oxford, Oxford OX1 3PU, UK; Institute of Functional Nano & Soft Materials (FUNSOM), Jiangsu Key Laboratory for Carbon-Based Functional Materials & Devices, State Key Laboratory of Bioinspired Interfacial Materials Science, Soochow University, Suzhou 215123, China; Institute of Functional Nano & Soft Materials (FUNSOM), Jiangsu Key Laboratory for Carbon-Based Functional Materials & Devices, State Key Laboratory of Bioinspired Interfacial Materials Science, Soochow University, Suzhou 215123, China; Institute of Functional Nano & Soft Materials (FUNSOM), Jiangsu Key Laboratory for Carbon-Based Functional Materials & Devices, State Key Laboratory of Bioinspired Interfacial Materials Science, Soochow University, Suzhou 215123, China; State Key Laboratory of Extreme Photonics and Instrumentation, College of Optical Science and Engineering; International Research Center for Advanced Photonics, Zhejiang University, Hangzhou 310027, China; Institute of Functional Nano & Soft Materials (FUNSOM), Jiangsu Key Laboratory for Carbon-Based Functional Materials & Devices, State Key Laboratory of Bioinspired Interfacial Materials Science, Soochow University, Suzhou 215123, China; Institute of Functional Nano & Soft Materials (FUNSOM), Jiangsu Key Laboratory for Carbon-Based Functional Materials & Devices, State Key Laboratory of Bioinspired Interfacial Materials Science, Soochow University, Suzhou 215123, China; Institute of Functional Nano & Soft Materials (FUNSOM), Jiangsu Key Laboratory for Carbon-Based Functional Materials & Devices, State Key Laboratory of Bioinspired Interfacial Materials Science, Soochow University, Suzhou 215123, China; Institute of Functional Nano & Soft Materials (FUNSOM), Jiangsu Key Laboratory for Carbon-Based Functional Materials & Devices, State Key Laboratory of Bioinspired Interfacial Materials Science, Soochow University, Suzhou 215123, China; Institute of Functional Nano & Soft Materials (FUNSOM), Jiangsu Key Laboratory for Carbon-Based Functional Materials & Devices, State Key Laboratory of Bioinspired Interfacial Materials Science, Soochow University, Suzhou 215123, China; Clarendon Laboratory, University of Oxford, Oxford OX1 3PU, UK; Institute of Functional Nano & Soft Materials (FUNSOM), Jiangsu Key Laboratory for Carbon-Based Functional Materials & Devices, State Key Laboratory of Bioinspired Interfacial Materials Science, Soochow University, Suzhou 215123, China; Institute of Functional Nano & Soft Materials (FUNSOM), Jiangsu Key Laboratory for Carbon-Based Functional Materials & Devices, State Key Laboratory of Bioinspired Interfacial Materials Science, Soochow University, Suzhou 215123, China; Clarendon Laboratory, University of Oxford, Oxford OX1 3PU, UK; Institute of Functional Nano & Soft Materials (FUNSOM), Jiangsu Key Laboratory for Carbon-Based Functional Materials & Devices, State Key Laboratory of Bioinspired Interfacial Materials Science, Soochow University, Suzhou 215123, China; Macao Institute of Materials Science and Engineering, Macau University of Science and Technology, Macau 999078, China

**Keywords:** quantum dots, near-infrared, light-emitting diodes, fluorophore, electroluminescence

## Abstract

Colloidal quantum dots (CQDs) are promising materials for constructing ‘second-window’ near-infrared (1000–1700 nm) light-emitting diodes (NIR-II LEDs), but their practical application has been hampered by low film external quantum efficiency (EQE). Here, we report a chemical strategy that incorporates photoactive fluorophores—spanning fluorescence, phosphorescence and thermally activated delayed fluorescence—into CQD films to boost NIR-II emission. Energy transfer from fluorophores (via both singlet and triplet pathways) raises the photoluminescence quantum efficiency of CQD to 85% beyond 1000 nm. As a result, these composite films power NIR-II LEDs with a record EQE of 25.3% for emission of >1000 nm, the highest among all LEDs with emission of >1000 nm. We further demonstrate the scalability of the approach by fabricating large-area (30 mm × 30 mm) NIR-II LEDs with uniform high performance.

## Teaser

Energy transfer from singlet and triplet fluorophores to quantum dots enables record-efficiency NIR-II electroluminescence, establishing a new strategy for high-performance near-infrared light-emitting devices.

## INTRODUCTION

Second-window near-infrared (NIR-II, 1000–1700 nm) light-emitting diodes (LEDs) promise transformative applications in night vision, optical communications and biomedical imaging [1–6]. While advanced materials—such as organic dyes and semiconducting polymers—have driven visible-light LEDs to external quantum efficiencies (EQEs) of >30%, they struggle in the NIR-II region (EQE < 5%) due to intrinsically low photoluminescence quantum efficiency (PLQE) governed by the energy-gap law. By reducing phonon coupling and enhancing wave-function overlap, quantum-confined semiconductors can mitigate non-radiative losses, making colloidal quantum dots (CQDs) attractive NIR emitters. To date, the highest reported EQE for NIR-II CQD-LEDs is ∼16%—barely half that of visible-emitting CQD-LEDs and far below that of GaN blue LEDs (∼80%) [7–11].

EQE is defined as the ratio of emitted photons to injected electron–hole pairs. The number of emitted photons depends on the fraction of injected charges that recombine radiatively and are coupled out of the device. Consequently, achieving a high EQE requires maximizing the PLQE of the CQD film. However, CQD films emitting >1000 nm typically exhibit low PLQEs due to prominent non-radiative recombination. Although recent work has reached an EQE of 20.5%, those devices are limited to emissions near 900 nm, indicating significant remaining scope for improvement in the >1000-nm region.

Efforts to prevent non-radiative recombination in CQD films have relied thus far on two major strategies: (i) quantum dot (QD) surface passivation by using a coating shell or functional ligands (Strategy-I) [12–18] and (ii) embedding CQDs within a continuous wide-bandgap matrix so that energy is funneled from the matrix into the QDs, increasing the radiative efficiency (Strategy-II) [9,19–21]. The Strategy-I platform has been used across a large color gamut, though a balance is required between improved surface passivation from strongly binding ligands and steric hindrance—including a coating shell that isolates the dots and increases the dot-to-dot distance—that reduces conductivity in films [12–17]. Strategy-II can improve charge transport in films and has been utilized in the highest-performance NIR-II QD-LEDs to date [9,19–21]. However, correct lattice matching and energetic alignment are required between the matrix and the QDs, heavily reducing the tunability of the system.

Here, we introduce a new strategy (Strategy-III) platform that leverages energy transfer from organic fluorophores integrated into CQD films—eliminating the need for lattice matching. We systematically evaluated fluorophores with fluorescent, phosphorescent and thermally activated delayed fluorescence (TADF) pathways. We discovered that embedding fluorescent fluorophores alongside CQDs dramatically enhances NIR-II PLQE via efficient Förster resonance energy transfer. Leveraging this strategy, we fabricated NIR-II LEDs that achieved a record EQE of 25.3%. This fluorophore–CQD composite platform opens a versatile route to high-efficiency NIR-II LEDs, paving the way for further optimization through tailored organic synthesis and device engineering.

## RESULTS

### Composite films

**Figure 1.**
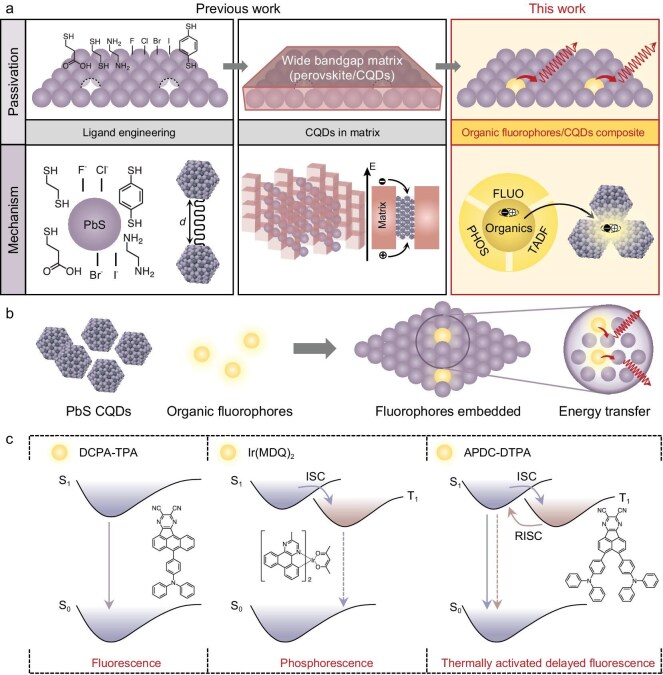
Photoactive fluorophores/CQD composite films for efficient luminescence. (a, b) Schematics demonstrating previous strategies to improve NIR luminescence efficiency: ligand exchange and dot-in-matrix strategies, along with fluorophore/CQD composite films. (c) Emission mechanisms for each of the fluorophores employed: fluorescence (emission primarily via the S_1_ → S_0_ transition), phosphorescence (enabled by the heavy-metal effect, which facilitates the otherwise forbidden transition from T_1_ → S_0_,) and TADF (utilizing a twisted donor–accepter structure to harvest both S_1_ and T_1_ states, ultimately emitting via the S_1_ → S_0_ transition), along with their corresponding chemical structures.

We first synthesized CQDs with emissions in the NIR-II region and screened three photoactive fluorophores with different emission mechanisms: singlet emission using the pure fluorescence of 8-(4-(diphenylamino)phenyl)aceanthryleno[1,2-b]pyrazine-2,3-dicarbonitrile (DCPA–TPA); triplet emission based on the phosphorescence of bis(2-methyldibenzo[f, h]quinoxaline) (acetylacetonate)-iridium(III) (Ir(MDQ)_2_); and delayed singlet emission based on the reverse intersystem crossing of 3,4-bis(4-(diphenylamino)phenyl)acenaphtho[1,2-b]pyrazine-8,9-dicarbonitrile (APDC–DTPA) (Fig. 1a–c). Importantly, each of these fluorophores has previously been demonstrated as the emission layer in an organic LED, the results of which we verified with our own devices (Figs S1–S3), suggesting potential compatibility in composite devices [22,23].

**Figure 2.**
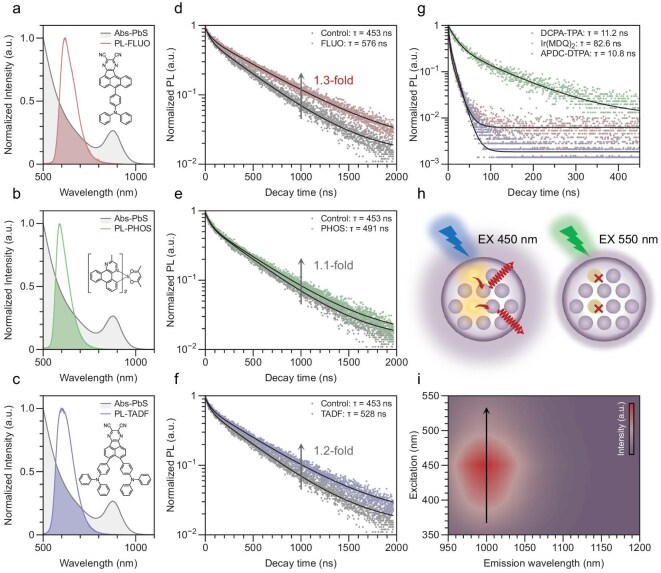
Photophysical spectroscopy of composite films. Absorption spectra of CQDs and photoluminescence spectra of (a) fluorescent, (b) phosphorescent and (c) TADF materials. Time-resolved photoluminescence (TRPL) spectra (455-nm excitation) of a control CQD film and composite films embedded with (d) fluorescent, (e) phosphorescent and (f) TADF materials. (g) TRPL of the pure fluorophore films. (h, i) Schematic and pseudocolor map of excitation-dependent PL of a composite film illustrating increased luminescence when excited at ∼450 nm. When the excitation wavelength is ∼450 nm, both the fluorophore and the QDs are excited and PL is enhanced. When excited at >500 nm (below the fluorophore bandgap), only the CQD is activated in the composite and the PL intensity reduces.

We measured the absorption of the control CQD films and photoluminescence (PL) from the three photoactive fluorophores separately to evaluate the possibility of energy transfer. The CQD film exhibited a well-defined absorption peak near 900 nm and the PL of the three photoactive fluorophores showed similar emission characteristics (emissions of ∼600–650 nm). The significant spectral overlap between these bands (Fig. 2a–c) confirms the presence of efficient energy-transfer pathways from the fluorophores to the CQDs, enabling both singlet and triplet exciton reutilization. Such overlap, together with a high radiative decay rate of the fluorophores, is therefore crucial for achieving efficient exciton harvesting and serves as a practical consideration when selecting fluorophores for NIR-II QD-LEDs.

To investigate further, we measured the PLQE of solutions and time-resolved PL (TRPL) of the NIR-II QD films with and without photoactive fluorophores (455-nm excitation) (Fig. 2d–f). The pure QD films (control) exhibited an average lifetime of ∼453 ns, consistently with the typical values reported for CQDs emitting in the NIR-II region. The fluorophore/QD composite films with the DCPA–TPA and the APDC–DTPA showed increased lifetime and higher PLQEs; the DCPA–TPA (singlet emission) showed the longest lifetime (∼576 ns)—1.3-fold compared with the value of the pure QD films and 1.1-fold compared with the value obtained in the APDC–DTPA (delayed singlet emission). These are consistent with PLQE increases from 61% to 82% (APDC–DTPA) and 85% (DCPA–TPA), with the emission peak position remaining unchanged. Embedding the Ir(MDQ)_2_ (triplet emission) had little effect, with a lifetime of 491 ns and PLQE of ∼60%.

Longer decay times and enhanced PLQEs suggest that fluorophores may bind to the QDs to passivate them or provide pathways for energy transfer between the photoactive components. To verify this, we conducted photoluminescence excitation measurements in which the PL spectra of control and composite films were collected under excitation at different wavelengths (Fig. 2h and i, and Fig. S4a). These results demonstrate that, when excited in the range of 400–500 nm, when both the fluorophore and the CQDs are excited, emission from the CQDs is boosted, suggesting that efficient energy transfer can occur in composite films. Femtosecond transient absorption spectroscopy supports this, as evidenced by a longer QD lifetime (2-fold longer) measured for fluorophore/NIR-II QD composite films, consistently with the TRPL and excitation-dependent PL results (Fig. S4b).

We studied the mechanism by analysing the photon-transfer process and calculating the rate of each process. In the DCPA–TPA (direct singlet emission), the electron jumps to an excited state S_1_ by energy absorption and then releases photons, with the molecule returning directly to the ground state S_0_. This process involves only one excited state and the rate of luminescence is fast (*k*_r_ = 8.87 × 10^7^ s^−1^), which enables a fast rate of energy transfer between photoactive fluorophores and NIR-II QDs [24]. For the Ir(MDQ)_2_ (triplet emission), although the use of a heavy metal enables the theoretically forbidden transition from triplet excited state T_1_ to the ground state S_0_, this is typically a slow process (*k*_r_ = 5.48 × 10^5^ s^−1^) and the pathways that compete with photo-recycling appear to result in less effective transfer. This also applies to the APDC–DTPA (delayed fluorescence), in which there is an additional spin-flipping process between the singlet excited state S_1_ and the triplet excited state T_1_. As the excitation of the electron is not direct, the rate of energy transfer is slower (*k*_r_ = 3.68 × 10^7^ s^−1^). Combining the mechanism analysis and the fact that the longest lifetime is from films containing the DCPA–TPA, we conclude that the exciton transfer rates are decisive to improve the probability of promoting NIR emission of the CQD composite films (Fig. S5).

### Repercussions in electronic devices

**Figure 3.**
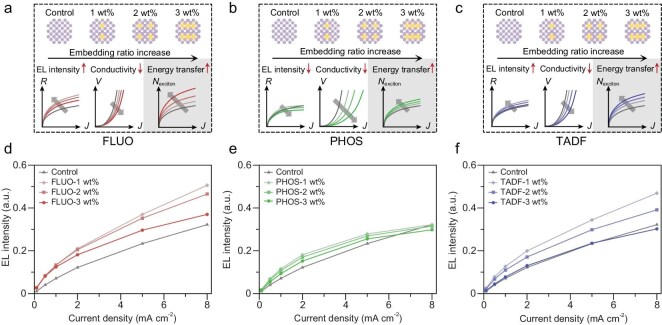
Electrical study of the composite films. Change in energy transfer, film conductivity and device performance due to the inclusion of photoactive fluorophores with different loading ratios. With (a) fluorescent, (b) phosphorescent and (c) TADF materials blended into the CQD film. EL intensity of devices with different loading ratios of (d) fluorescent, (e) phosphorescent and (f) TADF materials.

We then investigated the role of the fluorophores in real devices and determined the optimal fluorophore/CQD composite blend that maximizes radiative efficiency in the NIR-II LEDs (Fig. 3). To ensure reliability and reproducibility, we used the same device structure with the same CQD layer thickness in all devices to ensure that any changes in the current density–voltage and electroluminescence (EL) intensity measurements were the result of changes to the photoactive fluorophore ratio in each film. We tuned the weight ratio of the three photoactive fluorophores and observed a decreased current density when the fluorophore loading ratio was increased from 1 to 3 wt% for all three fluorophores (Fig. 3a–c). This could have originated from either the blocking of leakage paths in the CQD film, which would be a benefit, or reduced charge injection, due to the inability to effectively transport charge directly into the fluorophores, which would negatively affect the LED operation. Decreasing current density with increasing fluorophore loading suggested that there could be an optimum ratio at which the radiance is enhanced but the charge transport is not overly inhibited (Table S1). With further analysis, we found that the DCPA–TPA-embedded device (at 1 wt%) had a 1.8-fold higher EL intensity (1 W sr^−1^ m^−2^) compared with the control device, yet the current density demonstrated a continuous reduction with increasing ratio, representing an optimum balance between these competing processes (Fig. 3d–f and Figs S6–S9).

We also compared the effect of the photoactive fluorophores on film morphology, which is a prerequisite for fabricating high-performance LEDs. We characterized the film morphology of the photoactive fluorophores/CQD composite films in a range of 5 × 5 μm by using atomic force microscopy, with the pure CQD as the control. The pure CQD films appeared uniform, with a surface roughness *R*_q_ of 3.63 nm. For the composite films loaded with 1% wt of fluorophore, we measured *R*_q_ values of 2.76, 3.22 and 3.00 nm for the DCPA–TPA-, Ir(MDQ)_2_- and APDC–DTPA-embedded films, respectively (Fig. S10). The low roughness indicates that photoactive fluorophores were uniformly dispersed within the composite films without causing aggregation or an uneven surface distribution.

### NIR-II LEDs

**Figure 4.**
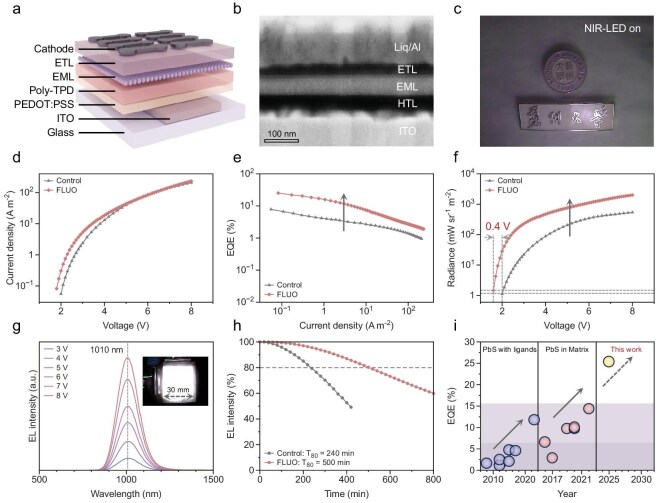
NIR-II QD-LED performance and stability. (a) Schematic diagram of ITO/PEDOT: PSS/Poly-TPD/fluorophores-CQD composite/ETL/Liq/Al LED device architecture. (b) Cross-sectional SEM image of a representative device. (c) Photograph taken via an NIR camera using emission from a NIR-QDLED. (d) *J*–*V* curves, (e) EQE–*J* curves and (f) *R*–*V* curves of the control and DCPA–TPA composite device. (g) Electroluminescence spectra of a DCPA–TPA composite NIR-II QD-LED with varying applied voltage. (h) Operational lifetimes of a control device and a DCPA–TPA composite device under the operating condition of 10 A m^−2^. (i) Summary of the EQEs achieved in the NIR-II for QD-LEDs using CQDs over time.

Leveraging this knowledge, we fabricated fluorophores/CQD composite NIR-II QD-LEDs. We used a device configuration of indium tin oxide (ITO)/poly(3,4-ethylenedioxythiophene)-poly(styrenesulfonate) (PEDOT: PSS)/poly[*N,N*'-bis(4-butylphenyl)-*N,N*'-bis(phenyl)-benzidine](Poly-TPD)/fluorophore–CQD composite/(1,3,5-triazine-2,4,6-triyl)tris(benzene-3,1-diyl)tris(diphenylphosphine oxide) (PO-T2T)/lithium 8-quinolate (Liq)/Al (Fig. 4a and Fig. S11). Cross-sectional scanning electron microscopy (SEM) images show that the various functional layers in the NIR-II QD-LEDs are clear and smooth (Fig. 4b).

The *J*–*V* curves show that the turn-on voltages of the three samples remain close to that of the control sample, with differences within 0.2 V (Fig. 4d). The EQE of the NIR-II QD-LEDs when using pure CQDs is 7.8%. The Ir(MDQ)_2_- and APDC–DTPA-embedded devices achieve higher EQEs of 18.7% and 15.4%, respectively, suggesting that the fluorophores can play a positive role (Fig. S12). Although the radiance of all three devices decreases with increasing embedding ratios, the DCPA–TPA-embedded devices benefit the most at level of 1 wt%, resulting in a maximum EQE of 25.3% (Fig. 4e and Fig. S13). The *R*–*V* curves show that the turn-on voltages remain close to that of the control sample, with differences within 0.4 V. Consistently with the previous analysis, the EL intensity is enhanced for the DCPA–TPA-embedded device, with a 3.7-fold increase in radiance compared with the control device (Fig. 4f). The NIR-II QD-LEDs exhibited a symmetric emission peak at 1010 nm. The peak position remained unchanged with a varying driving voltage, proving that the device had reasonable stability. To demonstrate the scalability of this method, we fabricated 30 × 30 mm^2^ large-area NIR-II QD-LEDs, in which the QD-solid film maintained uniform emission across a large area without any noticeable defects (Fig. 4g). Pictures taken under NIR-II lighting are crisp and clear, demonstrating that the NIR-II QD-LEDs can be operated at a reasonable radiance intensity (Fig. 4c).

Finally, we measured the operational stability of the fluorophore/CQD composite LEDs by applying a fixed current density of 10 A m^−2^ to a DCPA–TPA composite device. The T_80_ operating lifetime—the time at which the radiance decreased to 80% of its original—was 500 min, which was 2.1-fold longer than that of the device with QDs only (Fig. 4h).

## CONCLUSION

In summary, we introduce a new platform for enhancing CQD‐LED efficiency by blending photoactive fluorophores with CQDs to form composite films with improved NIR‐II luminance. We evaluated three fluorophore types—fluorescent (singlet), phosphorescent (triplet) and TADF—and demonstrated that energy transfer from fluorophores to CQDs significantly boosts CQD emission. NIR‐II QD-LEDs incorporating these composite films achieved a record EQE of 25.3% beyond 1000 nm (Fig. 4i). Our approach brings NIR‐II LED performance closer to visible‐range benchmarks and highlights the potential of optimized charge utilization to drive further breakthroughs. We anticipate that targeted synthesis and material blending will unlock even higher efficiencies, accelerating the adoption of NIR‐II LEDs in real‐world applications.

## Supplementary Material

nwaf552_Supplemental_File
