# Supplementary material for: Singlet and triplet harvesting enable efficient NIR-II quantum-dot electroluminescence
Source: Natl Sci Rev. 2025 Dec 4;13(3):nwaf552. doi: 10.1093/nsr/nwaf552 (PMC12866657; doi:10.1093/nsr/nwaf552)
Supplement: nwaf552_Supplemental_File [file nwaf552_supplemental_file.pdf]

SUPPORTING INFORMATION

Singlet and triplet harvesting enable efficient NIR-II quantum dot electroluminescence

Wan-Shan Shen[1,#], Sam Teale[2,#], Yang Liu[1], Jia-Lin Pan[1], You-Jun Yu[1], Chen Zou[3], Feng Zhao[1], Hong-Wei Duan[1], Ye Wang[1], Zong-Shuo Liu[1], Hua-Hui Li[1], Patrick Knowels[2], Zeke Liu[1], Ya-Kun Wang[1,*], Henry J. Snaith[2,*] and Liang-Sheng Liao[1,4,*]

[1]Institute of Functional Nano & Soft Materials (FUNSOM), Jiangsu Key Laboratory for Carbon-Based Functional Materials & Devices, State Key Laboratory of Bioinspired Interfacial Materials Science, Soochow University, Suzhou 215123, Jiangsu, China;

[2]Clarendon Laboratory, University of Oxford; Oxford OX1 3PU, UK;

[3]State Key Laboratory of Extreme Photonics and Instrumentation, College of Optical Science and Engineering; International Research Center for Advanced Photonics, Zhejiang University; Hangzhou 310027, Zhejiang, China;

[4]Macao Institute of Materials Science and Engineering, Macau University of Science and Technology; Taipa 999078, Macau, China

***Corresponding authors.** E-mails: wangyakun@suda.edu.cn; henry.snaith@physics.ox.ac.uk; lsliao@suda.edu.cn

[#]Equally contributed to this work

Experimental details

Materials: Poly[N,N'-bis(4-butylphenyl)-N,N'-bis(phenyl)-benzidine] (Poly-TPD) was purchased from Sigma-Aldrich. poly(3,4-ethylenedioxythiophene)-poly(styrenesulfonate) (PEDOT:PSS), (1,3,5-triazine-2,4,6-triyl)tris(benzene-3,1-diyl)tris(diphenylphosphine oxide) (POT2T), 1,3,5-tris(1-phenyl-1H-benzimidazol-2-yl)benzene (TPBi), lithium 8-quinolate (Liq), molybdenum trioxide (MoO_3), 4,4'-cyclohexylidenebis[N,N-bis(4-methylphenyl)aniline] (TAPC), tris(4-carbazoyl-9-ylphenyl)amine (TCTA), and bis(2-methyldibenzo[f,h]quinoxaline) (acetylacetonate) iridium(III) ($Ir(MDQ)_2$) were purchased from Xi'an Polymer Light Technology Corp. Lead acetate trihydrate ($PbAc_2 \cdot 3H_2O$, 99%) was purchased from Alfa Aesar. Hexane (Hex, 95%) and hexamethyldisilathiane (TMS-S, 98%) were purchased from J&K Scientific. All chemicals and reagents were used as received from commercial sources without further purification.

PbS QDs synthesis: The PbS QDs were synthesized via a rapid hot injection method by following the reported procedure. Briefly, 10 mmol of lead acetate trihydrate ($PbAc_2 \cdot 3H_2O$) and 7 g of oleic acid (OA) were dissolved in 60 g of 1-octadecene (ODE) in a three-neck flask by heating the mixture to 100°C under vacuum for 1 h. The sulfur precursor was prepared separately by mixing 1 mL of bis(trimethylsilyl)sulfide (TMS-S) and 9 mL of 1-octadecene. The reaction was initiated by rapid injection of the sulfur precursor into the lead precursor solution at 80°C under nitrogen. The QDs were cooled to room temperature slowly. The solution was then transferred into a nitrogen-filled glove box and purified by precipitation twice in hexane/isopropyl alcohol and once in hexane/acetone. The PbS QDs were stored with solid form in nitrogen-filled glove box.

OLED device fabrication: The devices were fabricated on pre-patterned indium tin oxide (ITO) glass substrates (150 nm thickness, sheet resistance: 16 Ω/square). Before device fabrication, ITO substrates were cleaned with ethanol, acetone, and deionized water, followed by drying at 120 °C for 2 h in a convection oven. Subsequently, the substrates were subjected to UV-ozone treatment for 25 min to enhance surface wettability and remove organic residues. Finally, the

cleaned substrates were transferred into a thermal evaporation chamber (Suzhou Fangsheng FS-300, based pressure: 3 × 10^{-6} Torr). OLEDs with the configuration of ITO/ molybdenum trioxide (MoO_3, 3 nm)/ 4,4'-cyclohexylidenebis[N,N-bis(4-methylphenyl)aniline] (TAPC, 40 nm)/ tris(4-carbazoyl-9-ylphenyl)amine (TCTA, 10 nm)/ EML (APDC-DTPA, $Ir(MDQ)_2$, or APDC-DTPA, 20 nm)/ 1,3,5-tris(1-phenyl-1H-benzimidazol-2-yl)benzene (TPBi, 60 nm)/ Liq (2 nm)/ Al (120 nm) were fabricated, respectively. Deposition rates and thicknesses of all materials were monitored with oscillating quartz crystals. The deposited rates for organic materials, Liq, and Al were controlled at 2, 0.2, and 6 Å s^{-1}, respectively. Each ITO substrate was patterned to yield four devices, each with an active area of 10 mm^2.

NIR-QDLED device fabrication: The patterned indium tin oxide (ITO) glass substrates were cleaned individually using ethanol and deionized water. Before device fabrication, the ITO substrates were treated with UV-ozone for 25 min. PEDOT:PSS was filtered through a 0.2 µm hydrophilic polytetrafluoroethylene (PTEE) syringe filter and spin-coated onto the prepared ITO substrate at 5,000 rpm for 60 s, followed by annealing at 150°C for 15 min in the air to remove the residual water. The following deposition processes were performed in the glovebox. Poly-TPD was spin-coated from an 8 mg mL^{-1} chlorobenzene solution onto PEDOT:PSS film at 3000 rpm for 30 s and then baked on a hot plate at 120°C for 10 min. After the substrate was cooled to room temperature, the as-synthesized PbS QDs were dispersed in hexane (20 mg mL^{-1}) and filtered through a polyvinylidene fluoride (PVDF) syringe filter for film by spin-coating at a spin rate of 3000 rpm for 120 s. Then the as-prepared PbS film was soaked in a 0.3% (volume ratio) solution of hexanedithiol (HDT) in acetonitrile (ACN) and rinsed with pure ACN by drop-casting onto the surface at 1000 rpm for 30 s to remove the free ligand molecules. The deposition procedure was repeated several times for dense emission layer at a target thickness. Subsequently, the substrate was moved into a thermal evaporation chamber (Suzhou Fangsheng FS-300, based pressure: 3 × 10^{-6} Torr). Furthermore, ETL (40 nm), Liq (2 nm), and Al (120 nm) layers were deposited at rates of 2, 0.2, and 6 Å s^{-1}, respectively. Each ITO substrate was patterned to yield four devices and each with an active area of 10 mm^2.

Film characterization and device measurements: The photoluminescence (PL) spectra were collected by a fluorescence spectrometer (NIR-VIS, FL3). PL decay spectra were obtained by using the PL-TCSPC fluorescence lifetime measurement system (C12132-38, Hamamatsu Photonics Co.) Visible-near-infrared absorption spectra of PbS QDs in hexane were recorded at room temperature using a UV-VIS-NIR spectrophotometer (Lambda950, PerkinElmer). Atomic force microscopy (AFM) measurements were operated by using a Cypher-S atomic force microscope (Asylum Research, Oxford Instruments). All the devices were characterized by ambient conditions. Current density-voltage (*J*–*V*) characteristics were monitored by using a computer-controlled Keithley 2400 source meter. Electroluminescence (EL) spectra, operational lifetime, and external quantum efficiency (EQE) were tested by using a calibrated photonic multichannel PMA-12 analyzer system (Hamamatsu C10027-01 (360-950 nm) and C10028-01 (950-1,600 nm)) for spectral shape acquisition, while the absolute radiance was measured by a PR-745 instrument (Photo Research, 380-1,060 nm).

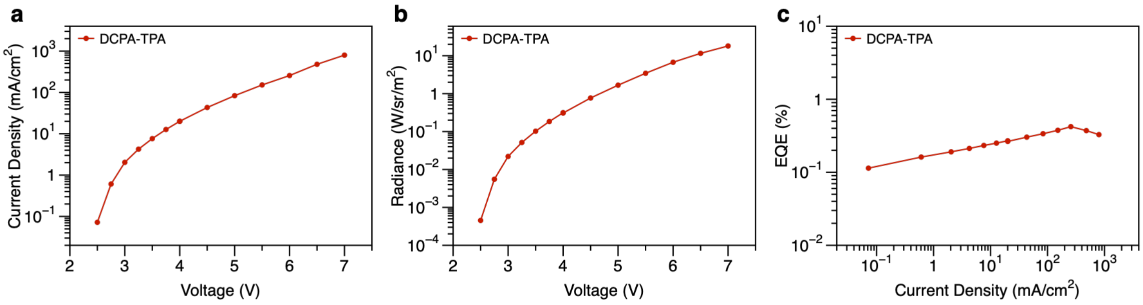

Figure S1. a, *J-V*, **b,** *R-V*, and **c,** *EQE-J* curves of DCPA-TPA-based OLEDs.

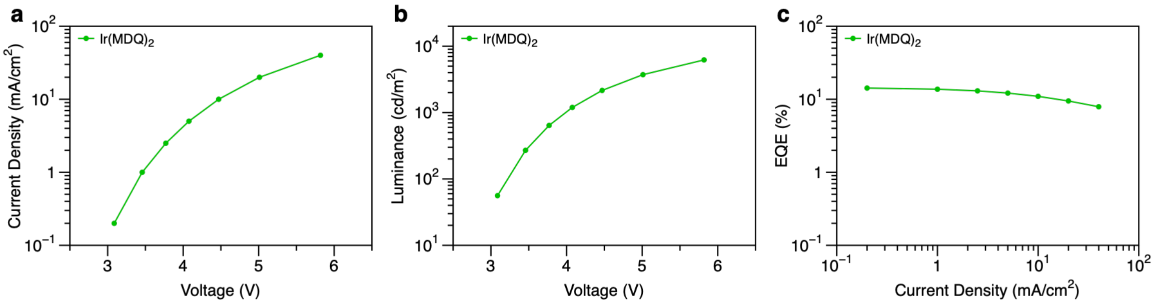

Figure S2. **a,** *J-V*, **b,** *L-V*, and **c,** *EQE-J* curves of Ir(MDQ)$_2$-based OLEDs.

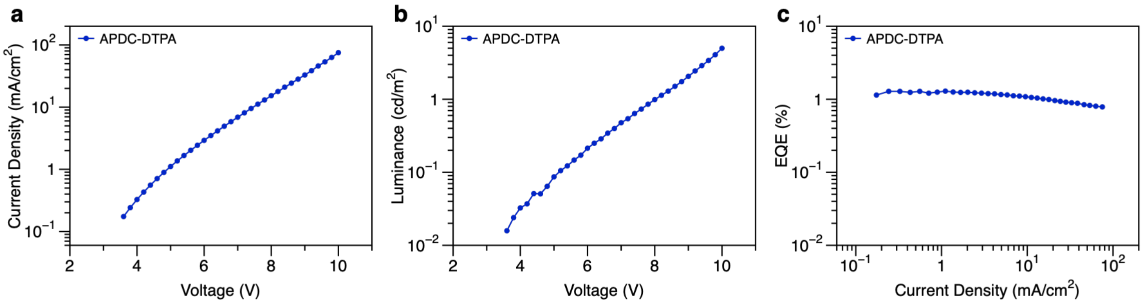

Figure S3. a, *J-V*, **b,** *R-V*, and **c,** *EQE-J* curves of APDC-DTPA-based OLEDs.

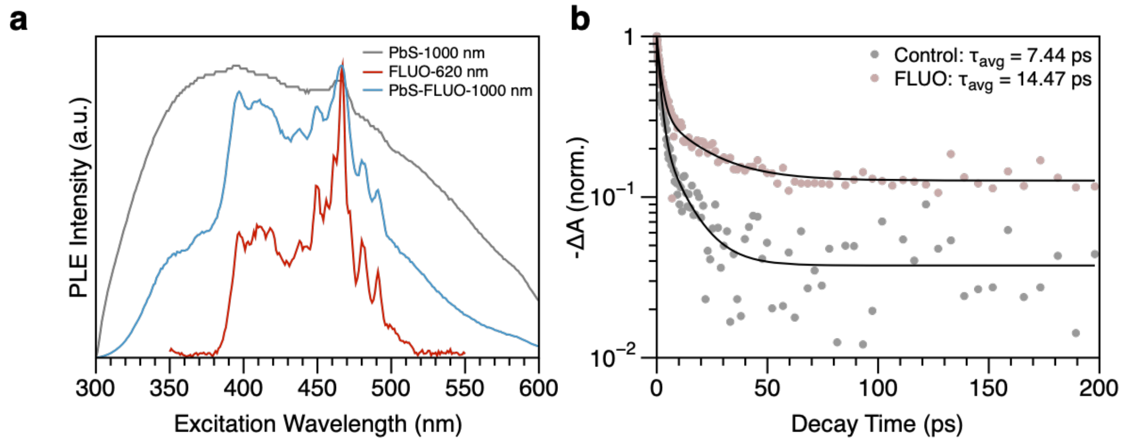

Figure S4. a, Photoluminescence excitation (PLE) spectra of pristine PbS CQDs (PbS-1000 nm, λ_{em} = 1000 nm), DCPA-TPA (FLUO-620 nm, λ_{em} = 620 nm), and DCPA-TPA/PbS CQDs composite (PbS-FLUO-1000 nm, λ_{em} = 1000 nm). **b,** TA curves of the pristine PbS CQDs (control) and DCPA-TPA/PbS CQDs composite (FLUO) (λ_{ex} = 400 nm, 270 fs, 50 kHz, 20 uJ/cm^2).

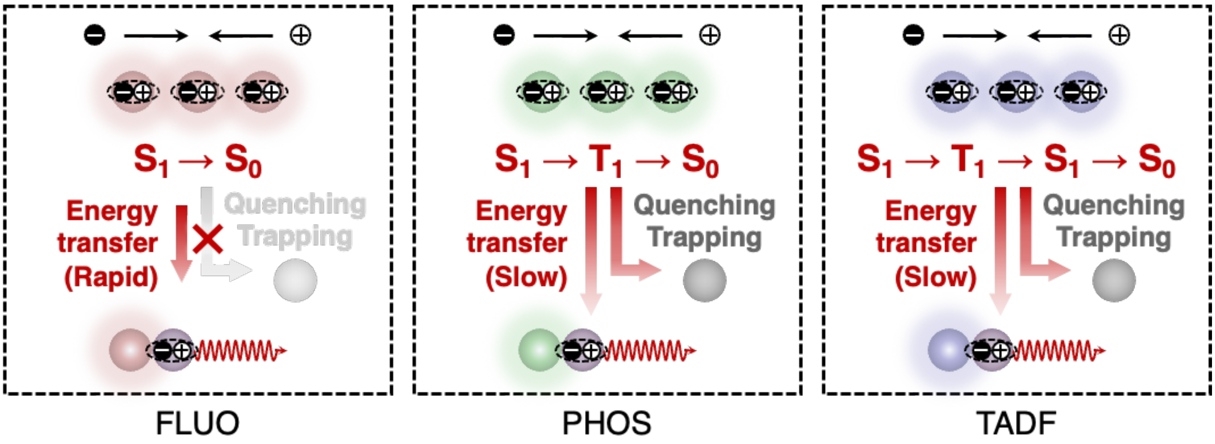

Figure S5. Summary of the energy transfer from fluorescent, phosphorescent, and TADF materials to PbS CQDs.

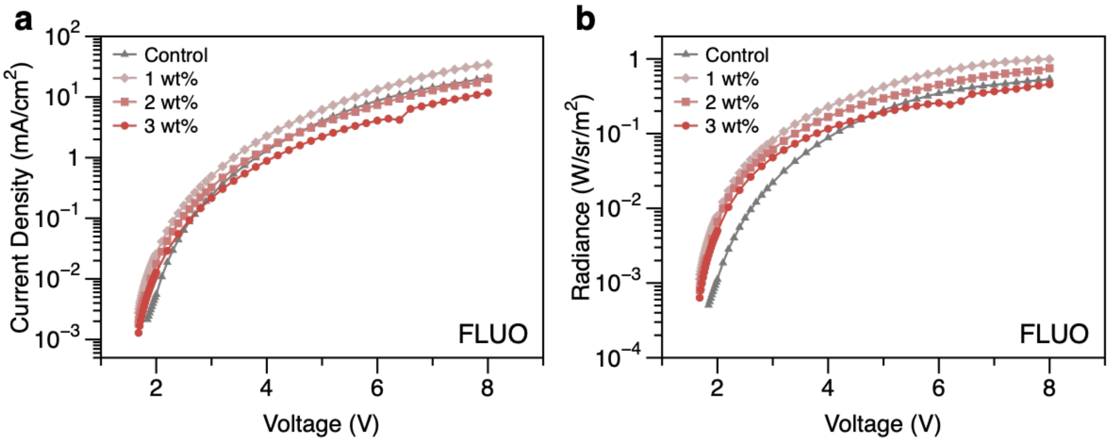

Figure S6. a, *J-V* and **b,** *R-V* curves of NIR-QDLEDs at different embedding ratios of DCPA-TPA.

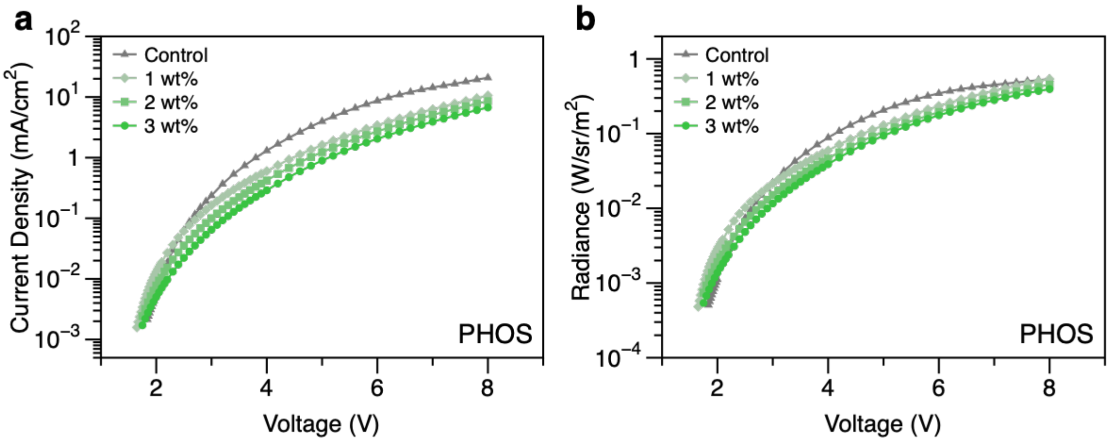

Figure S7. a, *J-V* and **b,** *R-V* curves of NIR-QDLEDs at different embedding ratios of $Ir(MDQ)_2$.

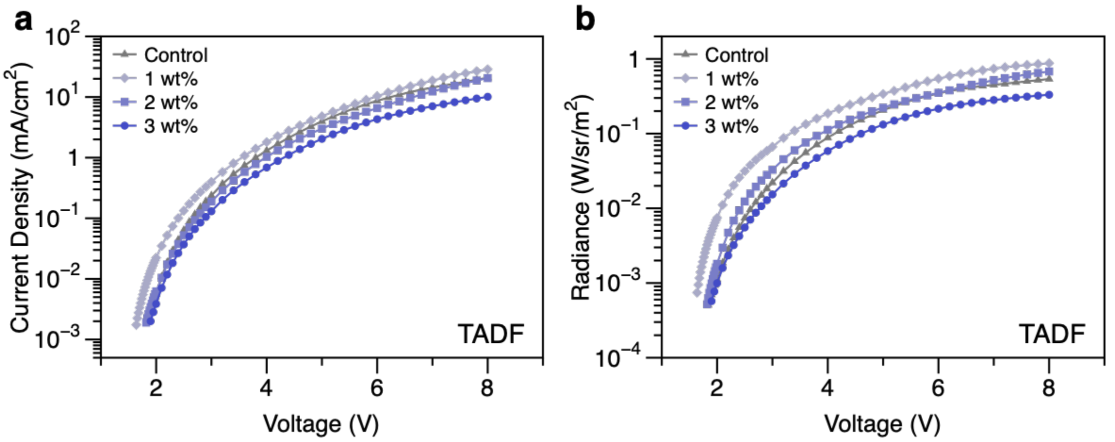

Figure S8. a, *J-V* and **b,** *R-V* curves of NIR-QDLEDs at different embedding ratios of APDC-DTPA.

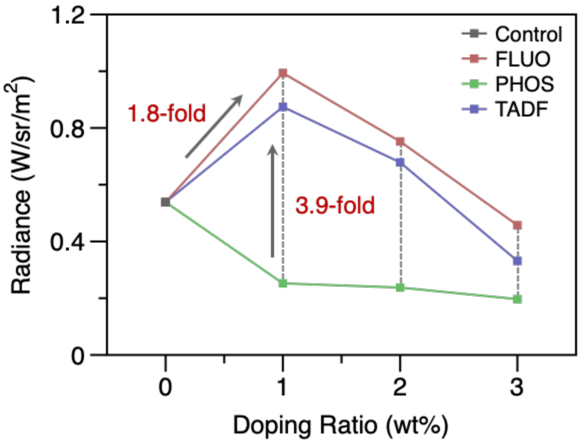

Figure S9. Radiance of composite NIR-QDLEDs with varying fluorophore embedding ratio.

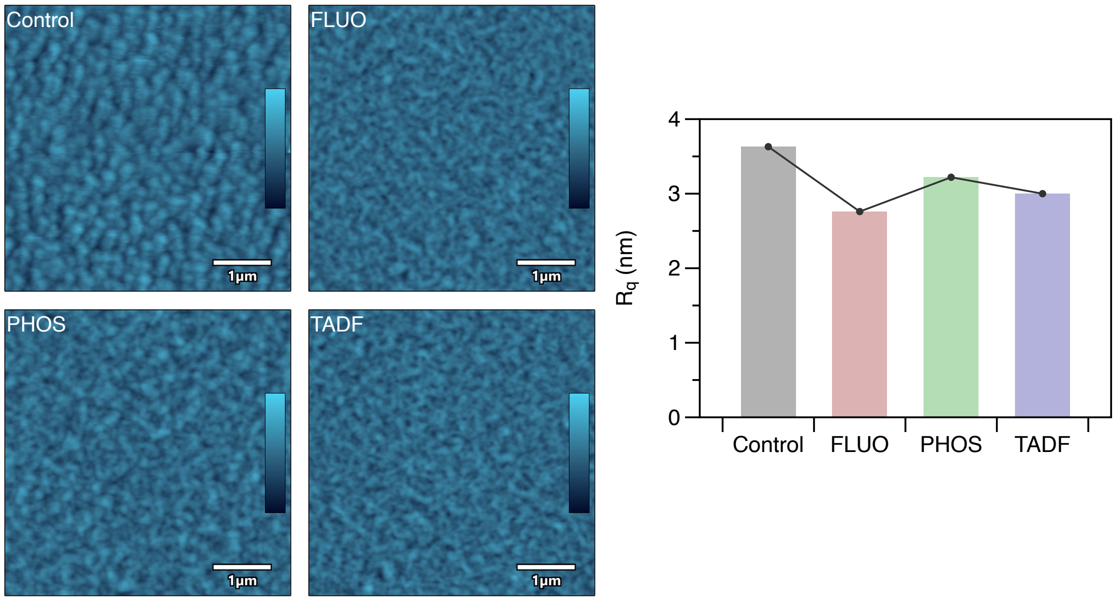

Figure S10. AFM roughness of the pristine PbS CQDs and the organic fluorophores/CQDs composite EML film embedded with DCPA-TPA, $Ir(MDQ)_2$, and APDC-DTPA, respectively.

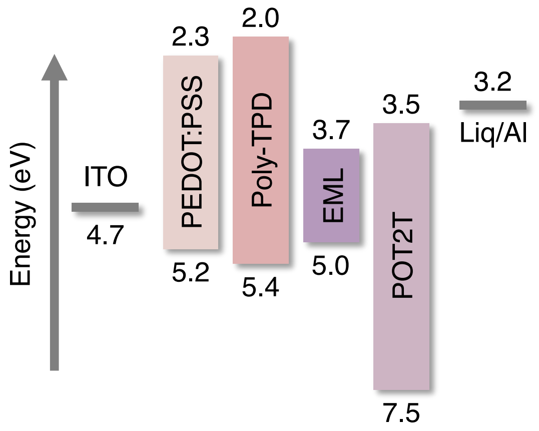

Figure S11. Energy level diagram of each layer in the ITO/PEDOT:PSS/Poly-TPD/EML/POT2T/Liq/Al device architecture.

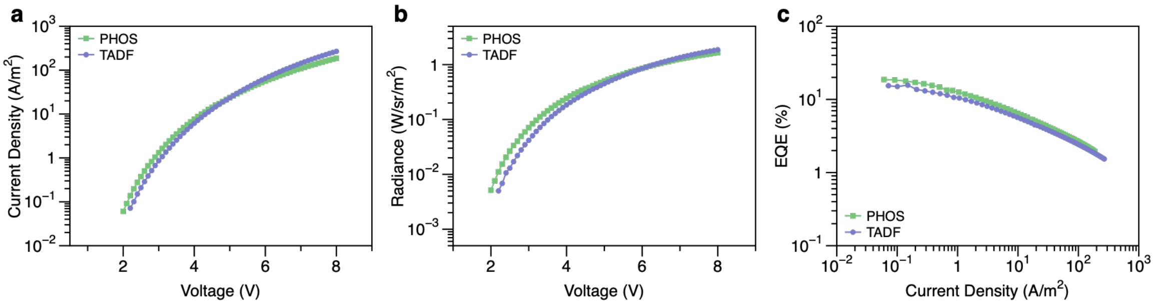

Figure S12. a, *J-V*, **b,** *R-V*, and **c,** *EQE-J* curves of Ir(MDQ)$_2$ and APDC-DTPA composite devices.

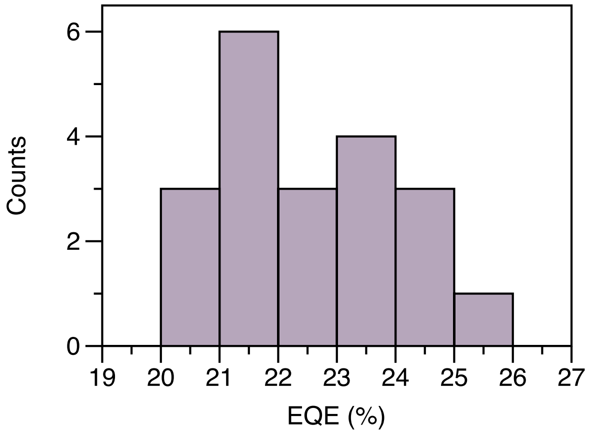

Figure S13. Histogram of the maximal EQEs extracted from the electroluminescence measurements of 20 independently fabricated devices.

	Ratio (wt%)	EL intensity (a.u.)					
		0.1 mA/cm^2	0.5 mA/cm^2	1 mA/cm^2	2 mA/cm^2	5 mA/cm^2	8 mA/cm^2
Control	-	0.011	0.040	0.071	0.122	0.233	0.322
FLUO	1	0.026	0.082	0.133	0.210	0.370	0.506
	2	0.027	0.083	0.133	0.205	0.352	0.465
	3	0.028	0.082	0.124	0.181	0.296	0.370
PHOS	1	0.018	0.069	0.116	0.181	0.279	0.322
	2	0.174	0.064	0.108	0.170	0.270	0.315
	3	0.015	0.055	0.093	0.152	0.256	0.298
TADF	1	0.026	0.078	0.127	0.199	0.344	0.469
	2	0.020	0.067	0.110	0.171	0.298	0.391
	3	0.012	0.045	0.078	0.130	0.235	0.302

Table S1. EL intensity at different current densities of the control and organic fluorophore/CQD composite sample.
